# Early Onset Parkinson’s Disease in a family of Moroccan origin caused by a p.A217D mutation in *PINK1*: a case report

**DOI:** 10.1186/s12883-017-0933-z

**Published:** 2017-08-08

**Authors:** Brendan P. Norman, Steven J. Lubbe, Manuela Tan, Naomi Warren, Huw R. Morris

**Affiliations:** 1 Department of Clinical Neuroscience, Institute of Neurology, University College London, London, UK; 2 Ken and Ruth Davee Department of Neurology, Northwestern University Feinberg School of Medicine, Chicago, USA

**Keywords:** Early Onset Parkinson’s Disease, *PINK1* p.A217D

## Abstract

**Background:**

Bi-allelic mutations in the genes *Parkin* (PARK2), *PINK1* (PARK6) and *DJ-1* (PARK7) are established causes of autosomal recessive early-onset Parkinson’s Disease (EOPD). *PINK1* mutations are the second commonest cause of EOPD. Specific mutations may be relatively common in certain populations because of a founder effect. Homozygous p.A217D *PINK1* mutations were previously shown to cause EOPD in a large Sudanese kindred.

**Case presentation:**

Here we report the segregation of homozygous *PINK1* p.A217D mutations in a family originating in Morocco with a history of parental consanguinity. From the clinical information available for the index case, the phenotype of mild, slowly-progressive Parkinsonism is consistent with previous reports of p.A217D disease and of *PINK1* disease phenotype more generally. The reported features of early prominent lower-limb symptoms and gait disturbance with asymmetrical onset are more frequent among *PINK1* disease cases.

**Conclusions:**

Together, reports of p.A217D in families of Moroccan and Sudanese origin suggest that p.A217D is a North African mutation due to a founder effect. Wider genetic analyses of EOPD in North Africa would be useful to estimate the prevalence of Parkinsonism caused by *PINK1* p.A217D. In the absence of bi-allelic *Parkin* mutations, *PINK1* mutations should be considered in cases with evidence of autosomal recessive inheritance of EOPD and presentation of atypical features such as early lower-limb symptoms and gait disturbance with asymmetrical onset, which appear to be common in Mendelian EOPD.

## Background

Parkinson’s Disease (PD) is the second commonest neurodegenerative disease, affecting approximately 1% of people over 50 years of age [1]. PD is caused by progressive selective death of dopaminergic neurons of the substantia nigra pars compacta [2].

Although most PD is sporadic, the discovery of genetic variants linked with PD in rare families with multiple affected members has elucidated some of the pathogenic mechanisms underlying neurodegeneration. In early-onset Parkinson’s Disease (EOPD), genetic factors are probably more prominent, as there is a consistently stronger effect of familial aggregation compared to typical late onset PD [3]. Increased disease risk for siblings of people affected by EOPD as compared to parents is consistent with autosomal recessive disease.

Mutations in PARK2 (*Parkin*), PARK6 (*PINK1*) and PARK7 (*DJ-1*) cause autosomal recessive EOPD. *Parkin* point mutations and deletions are the commonest genetic cause of EOPD, with worldwide prevalence of 8.6% in EOPD. *PINK1* mutations have intermediate prevalence of 3.7%, while *DJ-1* mutations are rarer, accounting for 0.4% of EOPD [4]. Causal mutations in *Parkin*, *PINK1* and *DJ-1* implicate disrupted mitochondrial homeostasis as a key pathophysiologic event in EOPD. Amongst other diverse functions, *Parkin* and *PINK1* have a well-established interactive role in mitochondrial quality control [5–7].

Over 40 point mutations and rarer large deletions in *PINK1* are reported to be pathogenic when inherited as bi-allelic mutations in EOPD cases [8]. *PINK1* mutation prevalence varies with ethnicity, with greater prevalence in Asian cases (13.5% among EOPD cases) compared to white or Latin American cases (<1% among EOPD cases) [4]. Despite the large number of different *PINK1* mutations reported, individually each is rare [9]. The pathogenicity of rare mutations shown to segregate with EOPD in just one family is less certain. Identification of families showing segregation of the same bi-allelic variant increases confidence in its pathogenicity. Here we report a new family with *PINK1* disease.

## Case presentation

### Patients

**Fig. 1:**
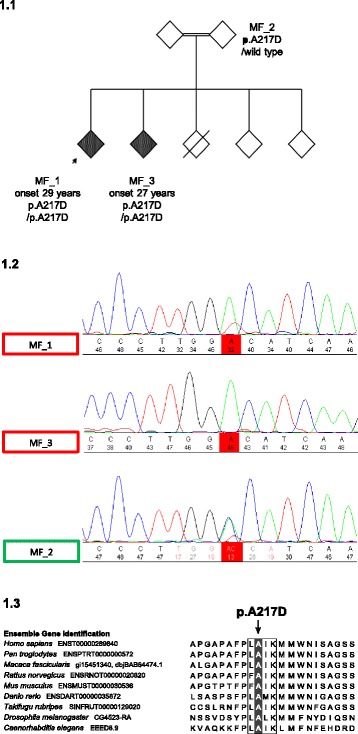
**a** Genetic pedigree of the family, with EOPD status indicated by shaded diamonds. Arrow indicates index case. **b** Chromatographs from sequencing with the c.650 position of *PINK1* exon 2 highlighted. Traces for affected family members MF_1 (index case) and MF_3 show homozygous C > A transversions, while that of the ‘carrier’ MF_2 shows a heterozygous transversion. **c** Multiple alignments of the *PINK1* protein in clustal format. Box indicates highly conserved ATPase orientation site. Shading indicates the position of the p.A217D mutation

The patients were of French North African descent, originating in Morocco. The siblings were born in France. The kindred comprises two generations, of which two second-generation family members had a Parkinsonian disorder (Fig. 1a). One second-generation family member died in 2006 in a road traffic accident. The parents were first cousins.

DNA was available for four family members: two affected (MF_1 [index] and MF_3) and one unaffected (MF_2).

### Clinical history

Clinical information was only available for the index case (MF_1), who developed symptoms in 2003, age 29 years with tremor of the left hand, micrographia, slowness in walking and a sensation of heavy legs. In 2005, symptoms progressed, with a tendency to drag the right leg. MF_1 was first examined in 2009, age 34 and had bilateral bradykinesia, rigidity, postural and rest tremor. Tremor and rigidity were noticeably worse on the left. Additional features included mild facial hypomimia, lower-limb hyper-reflexia and eye movement ‘catch-up’ saccades without noticeable slowness of eye movements in the vertical plane. A DaTscan showed reduced dopamine receptor binding in the putamen bilaterally consistent with nigrostriatal cell loss. There were no signs of cognitive nor neuropsychiatric disturbance, and tests for copper abnormalities, neuroferritinopathy and adjusted calcium, liver and thyroid function were normal. An MRI brain scan revealed no significant abnormality. MF_1 reported three falls within a twelve month period but was independent in activities of daily living. Symptoms were mild in the morning with deterioration as the day progressed. MF_1 was prescribed rasagiline, with some improvement in gait.

Six years after symptom onset a formal detailed assessment was carried out. Motor assessment: MF_1 completed parts II (‘motor aspects of experiences of daily living’) and III (‘motor examination’) of the Movement Disorder Society revised Unified Parkinson’s Disease Rating Scale (MDS-UPDRS; [10]) and recorded total scores of 7/52 and 38/429 respectively. Notable findings from the part III motor examination were increased bradykinesia and rigidity for the lower body compared with upper body, with greater impairment on the left side. Hoehn and Yahr stage was 2; classified as ‘bilateral impairment without impairment of balance’ [11].

Neuropsychological assessment: MF_1’s scores were both within the defined ‘normal’ range for both the Montreal Cognitive Assessment (MoCA; [12]), Mini-Mental State Examination (MMSE; [13]) and Geriatric Depression Scale-15 [14].

Non-Motor Assessment: Scores for the Epworth Sleepiness Scale [15] and overall sleep quality (Pittsburgh Sleep Quality Index [16]) were normal. Responses to the Non-Motor Symptoms Questionnaire (NMS-Quest; [17]) did not indicate any significant abnormality of sleep, sphincter or other autonomic function and there was no significant postural drop in blood pressure.

In 2011 MF_1 reported making some typing mistakes at work due to bradykinesia and reported some back and shoulder pain. In 2012, symptoms had progressed, with a gradual increase in slowness of movement and rigidity. Tremor was mild, but speech had deteriorated, with more noticeable dysarthrophonia. Symptoms responded well to ropinirole, which MF_1 had been taking for around six months at the time of examination. In 2012, MF_1 was continuing to work full-time and walk to work every morning. MF_1 was not taking L-DOPA.

### Genetic analysis

Genetic analysis of the family was approved under the Research Ethics Committee for Wales (Wales REC; 14/WA/1179; Clinical Neurological Disease Bio-bank and Neurogenetics Research Study 2 (CANDAS2)). Diagnostic testing was performed for *Parkin* and *LRRK2* only. Whole exome sequencing was sought on the index case to eliminate other established recessive mutations. Briefly, sample libraries were prepared using Illumina capture kits with paired-end sequencing performed on the Illumina HiSeq2000. All reads were aligned using BWA against the UCSC hg19 reference genome [18]. Variant calling and quality-based filtering were done using GATK [19]. ANNOVAR [20] was used to annotate variants. Sanger sequencing was performed to validate any identified variants.

Initial genetic tests of the index case did not find a *Parkin* or *LRRK2* mutation. Subsequent whole exome sequencing confirmed absence of *Parkin* and *LRRK2* mutations, but revealed a homozygous p.A217D mutation in *PINK1* (rs74315360).

Sanger sequencing confirmed the p.A217D mutation (Fig. 1b). Cases show homozygous C > A transversions at c.650 (NM_032409.2:c.650C > A), resulting in an alanine to aspartic acid substitution at position 217 of PINK1. The unaffected family member analysed shows a heterozygous transversion, identifying them as a carrier of the p.A217D mutation. We have therefore identified the genetic cause of this family’s EOPD.

## Discussion and conclusions

Although *PINK1* mutations are an established cause of EOPD, to our knowledge this is the second report of the *PINK1* p.A217D mutation. p.A217D is a missense mutation altering a highly-conserved amino acid of the kinase domain in the ATPase orientation site of *PINK1* (Fig. 1c). p.A217D was predicted to be damaging by all six *in silico* prediction tools from the genetic analysis [21–26]. Homozygous *PINK1* p.A217D mutations were previously found to segregate with EOPD in a large Sudanese kindred, in addition to its absence from 394 sequenced control chromosomes [27]. The rarity of this mutation is confirmed by its absence from the ExAC database (http://exac.broadinstitute.org), comprising approximately 60,000 individuals, of which about 5000 are African or African-American, likely to be of largely sub-Saharan origin [28]. The finding that the homozygous p.A217D mutation segregates with PD in this family of Moroccan origin adds weight to its pathogenic status.

Similarities between the clinical phenotype of the index case here and that previously reported for homozygous p.A217D PD cases are classical Parkinsonism and a relatively benign disease course. The most prominent difference is the later age at onset (AAO) of the cases here (27–29 years) compared to the previous p.A217D cases’ mean AAO of 12.6 years (range: 9–14 years).

The clinical phenotype reported here is consistent with the documented *PINK1* EOPD phenotype of relatively mild, slowly progressive classical Parkinsonism [29–32]. An interesting feature of the index case’s disease here is early, asymmetric gait disturbance. Lower limb symptoms and particularly gait disturbance are typically late features of idiopathic PD and usually accompanied by motor and cognitive decline [33] but they appear to be prominent early features of *PINK1* disease [27, 34–36]. The index case showed prominent lower limb hyper-reflexia upon examination; hyper-reflexia has been reported to be more frequent in *PINK1* cases than cases without *PINK1* mutations or cases with *Parkin* mutations [32].

The literature on *PINK1* EOPD suggests increased prevalence of some non-motor PD symptoms compared with idiopathic PD. These include mild cognitive impairment [37–41] and psychiatric abnormalities such as affective and behavioural disorders [34–36, 42, 43]. However prominent autonomic features, common in Lewy body PD such as REM sleep behaviour disorder, constipation and urinary symptoms are usually absent in *Parkin* and *PINK1* disease. The index case in this study did not have significant cognitive, neuro-psychiatric or autonomic clinical features. In summary, EOPD cases with homozygous *PINK1* p.A217D mutations reported here *a)* share most clinical features with previously reported p.A217D cases except with older AAO, *b)* are consistent with the previously reported *PINK1* EOPD phenotype more generally but with earlier AAO and without cognitive or neuropsychiatric disturbance, and together with other reports support the presence of features that add to a unique clinical Parkinsonian phenotype associated with *PINK1* mutations.

As the second report of p.A217D in cases originating in North Africa, p.A217D is probably a North African *PINK1* mutation. Specific *PINK1* mutations have relatively high frequency in some populations. For example the p.L347P mutation has a carrier frequency of 8% in the Philippines, indicating a founder effect [44]. Genetic analysis is not widely performed in North Africa, so further patients and kindreds may be found with *PINK1* p.A217D disease.

In conclusion, we report the second occurrence of the *PINK1* p.A217D mutation in a family affected by EOPD. Our result adds weight to the pathogenic status of this mutation and expands knowledge of the geographic diversity of p.A217D mutations to the Moroccan sub-region of North Africa.

## Abbreviations

AAO: Age at onset
EOPD: Early-onset Parkinson’s Disease
PD: Parkinson’s Disease
